# Obesity does not promote tumorigenesis of localized patient-derived prostate cancer xenografts

**DOI:** 10.18632/oncotarget.10258

**Published:** 2016-06-23

**Authors:** Jennifer C.Y. Lo, Ashlee K. Clark, Natasha Ascui, Mark Frydenberg, Gail P. Risbridger, Renea A. Taylor, Matthew J. Watt

**Affiliations:** ^1^ Metabolic Disease and Obesity Program, Monash Biomedicine Discovery Institute, Department of Physiology, Monash University, VIC 3800, Australia; ^2^ Monash Partners Comprehensive Cancer Consortium and Cancer Program, Monash Biomedicine Discovery Institute, Prostate Cancer Research Group, Departments of Physiology and Anatomy and Developmental Biology, Monash University, VIC 3800, Australia

**Keywords:** prostate cancer, obesity, human, xenograft, periprostatic adipose tissue

## Abstract

There are established epidemiological links between obesity and the severity of prostate cancer. We directly tested this relationship by assessing tumorigenicity of patient-derived xenografts (PDXs) of moderate-grade localized prostate cancer in lean and obese severe combined immunodeficiency (SCID) mice. Mice were rendered obese and insulin resistant by high-fat feeding for 6 weeks prior to transplantation, and PDXs were assessed 10 weeks thereafter. Histological analysis of PDX grafts showed no differences in tumor pathology, prostate-specific antigen, androgen receptor and homeobox protein Nkx-3.1 expression, or proliferation index in lean versus obese mice. Whilst systemic obesity per se did not promote prostate tumorigenicity, we next asked whether the peri-prostatic adipose tissue (PPAT), which covers the prostate anteriorly, plays a role in prostate tumorigenesis. *In vitro* studies in a cellularized co-culture model of stromal and epithelial cells demonstrated that factors secreted from human PPAT are pro-tumorigenic. Accordingly, we recapitulated the prostate-PPAT spatial relationship by co-grafting human PPAT with prostate cancer in PDX grafts. PDX tissues were harvested 10 weeks after grafting, and histological analysis revealed no evidence of enhanced tumorigenesis with PPAT compared to prostate cancer grafts alone. Altogether, these data demonstrate that prostate cancer tumorigenicity is not accelerated in the setting of diet-induced obesity or in the presence of human PPAT, prompting the need for further work to define the at-risk populations of obesity-driven tumorigenesis and the biological factors linking obesity, adipose tissue and prostate cancer pathogenesis.

## INTRODUCTION

Prostate cancer is the most common cause of cancer-related deaths globally and the incidence of prostate cancer has increased over the last two decades to be the second most commonly diagnosed cancer for males [1, 2]. The incidence of obesity has significantly increased during the same time, such that two in three men are now defined as overweight or obese [3]. While obesity is a major risk factor for many cancers, accounting for approximately one-third of cancer related deaths in 2012 [4], the epidemiological evidence linking obesity to prostate cancer incidence and disease outcomes is conflicting. Obesity has a mild or no association with prostate cancer incidence [5] but increases the risk of being diagnosed with advanced, high-grade prostate cancer [Relative Risk: 1.14 95% CI: 1.04–1.25] [6], increases biochemical recurrence after primary treatment [Relative Risk: 1.21 95% CI: 1.11–1.31] [5] and increases prostate cancer-specific mortality [Relative Risk: 1.15 95% CI: 1.06–1.25] compared to men of a healthy weight [5]. Thus, the weight of epidemiologic evidence suggests that obesity promotes aggressive prostate cancer, and provides a sound rationale to directly examine this association.

Murine studies support a pro-tumorigenic role for obesity in prostate cancer. Tumor mass and proliferation within xenografts derived from immortalized human prostate cancer cells (e.g. PC3 or LNCaP) are generally increased in mice rendered obese by high-fat feeding compared with lean mice fed a low-fat diet [7–10], while diet-induced obesity accelerates prostate cancer progression /aggressiveness in transgenic mouse strains of prostate cancer (e.g. TRAMP, Hi-Myc, *Pten*^−*/+*^) [9–15]. While these data are convincing, they are limited to immortalized metastatic cell lines and genetically modified mice, and studies using additional models that more closely replicate the biology of human prostate cancer are required to confirm these initial observations. In addition, these models do not faithfully mimic the structure/function relationship of the human prostate, which is covered anteriorly by a prominent peri-prostatic adipose tissue (PPAT). PPAT mass is increased in obese humans and is a risk factor for high-grade disease [16, 17], and the secreted factors from human PPAT are reported to promote tumorigenicity in cultured cells [18, 19]. Hence, PPAT is likely to be functionally relevant for prostate cancer progression.

Given that ~80% of prostate cancer patients have localized disease [20], we asked whether obesity presents a greater risk of developing an aggressive form of prostate cancer. Herein, we have utilized our well-established patient-derived xenograft (PDX) model [21] to address two clinically relevant inter-related questions: does obesity promote progression in moderate-grade localized human prostate cancer and/or, is the local impact of PPAT important in the tumorigenic process?

## RESULTS

### High-fat feeding recapitulates the features of an obese phenotype in SCID mice

**Figure 1:**
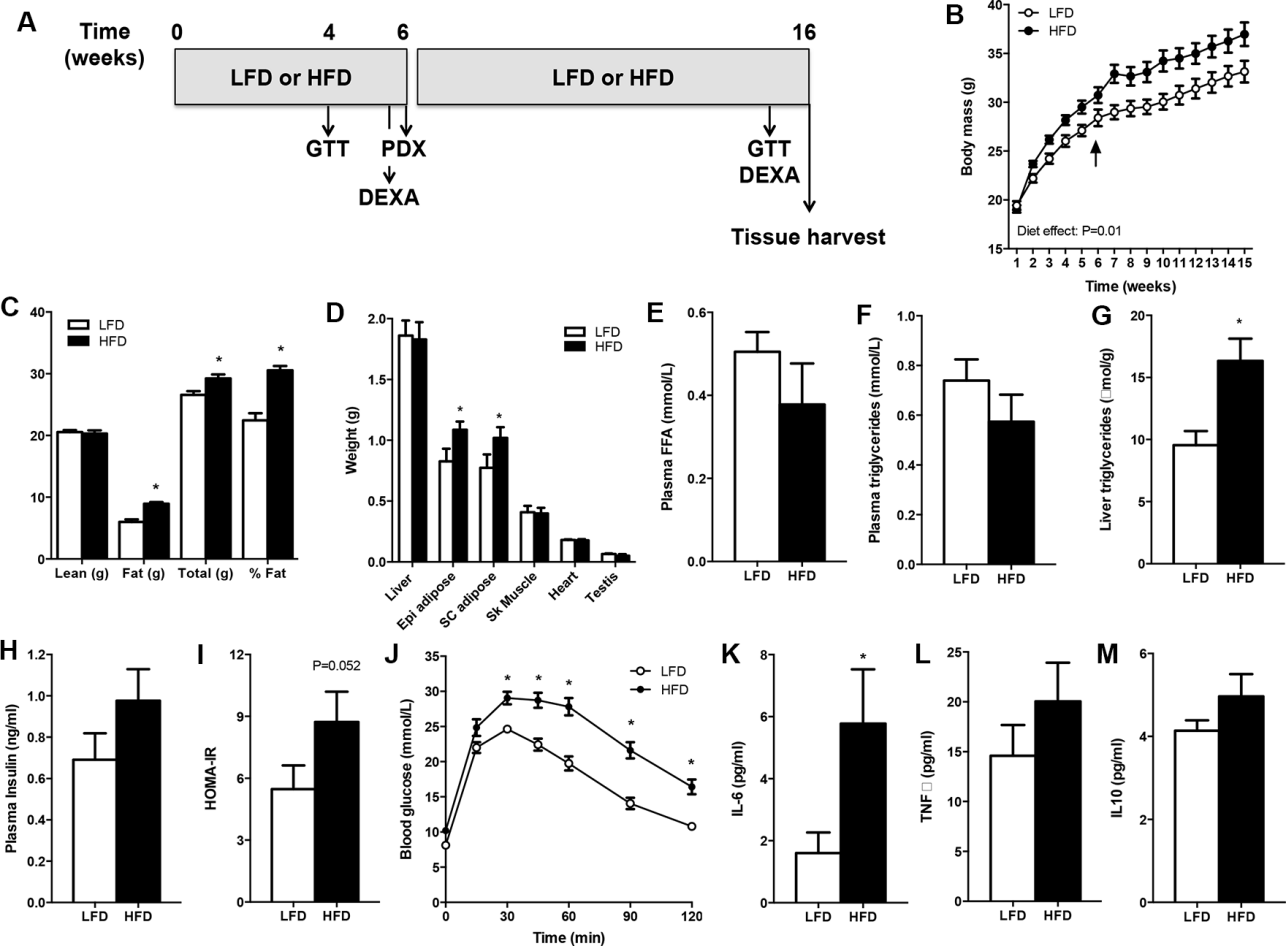
SCID mice develop diet-induced obesity Mice were fed a low-fat (LFD) or high-fat diet (HFD) for 16 weeks. (**A**) Schematic of the study design. (**B**) Body mass. Arrow denotes PDX surgery. *n* = 10 for LFD, *n* = 9 for HFD. (**C**) Body composition assessed by DEXA. *n* = 10 for LFD, *n* = 9 for HFD. (**D**) Tissue weights of mice. *n* = 9 for LFD, *n* = 10 for HFD. (**E**) Plasma free fatty acid (FFA) concentration. *n* = 7 for LFD, *n* = 6 for HFD. (**F**) Plasma triglyceride concentration. *n* = 9 for LFD, *n* = 7 for HFD. (**G**) Liver triglyceride content. *n* = 9 for LFD, *n* = 7 for HFD. (**H**) Plasma insulin concentration. *n* = 7 for LFD, *n* = 6 for HFD. (**I**) Insulin sensitivity calculated by the homeostatic model assessment (HOMA). *n* = 7 for LFD, *n* = 6 for HFD. (**J**) Glucose tolerance of mice. *n* = 12 for LFD, *n* = 12 for HFD. (**K**) Plasma IL-6. *n* = 10 for LFD, *n* = 9 for HFD. (**L**) Plasma TNFa. *n* = 9 for LFD, *n* = 6 for HFD. (**M**) Plasma IL-10. *n* = 10 for LFD, *n* = 9 for HFD. Data presented in panel C was derived from mice aged 6 weeks, data in panels D-M was derived in mice aged 15–16 weeks. *P < 0.05 HFD *vs.* LFD. Statistical analysis for panels B and I was by two-way repeated measures ANOVA with Bonferroni post hoc testing. For panels C–H, data were compared by unpaired two-tailed *t*-tests. Data are presented as the mean ± SEM.

SCID mice had similar body masses before allocation to the LFD or HFD (Figure 1B). Mice fed the HFD had an increased body mass compared with the LFD group at the time of surgery (Figure 1B, denoted by arrow), and this was entirely accounted for by a 48% increase in fat mass (Figure 1C). Body mass was greater in HFD *vs.* LFD mice throughout the period after PDX. Fat mass, but not lean mass (e.g. liver, heart, skeletal muscle), was different between groups after 15 weeks of high-fat feeding (Figure 1D), demonstrating the efficacy of the dietary regime to induce obesity.

While serum free fatty acids (Figure 1E) and triglyceride (Figure 1F) levels were not different between groups, liver triglyceride content was increased by 216% in HFD *vs*. LFD mice (Figure 1G), demonstrating hepatic steatosis, which is a prominent comorbidity of obesity. Fasting serum insulin was increased by 41% (*P* = 0.08, Figure 1H) and the homeostatic model assessment of insulin resistance was increased by 60% (Figure 1I) in HFD *vs.* LFD mice, indicating that mice fed the HFD had impaired insulin sensitivity compared with LFD mice. Furthermore, HFD mice were remarkably glucose intolerant compared with littermates fed a LFD (Figure 1J). Obesity is often described as a low-grade inflammatory state and consistent with this notion plasma IL-6 was increased, and TNFa (*P* = 0.14) and IL-10 (*P* = 0.08) tended to increase, in obese vs. lean mice (Figure 1K, 1L, 1M). Collectively, these results indicated that the HFD created an obesogenic environment before xenograft implantation and that this was maintained throughout the grafting period.

### High-fat feeding does not promote tumorigenesis in patient-derived xenografts grown in obese SCID mice

All patients had moderate-grade prostate cancer based on Gleason grade, prostate-specific antigen (PSA) and pathological stage (Table 1). We used immunohistochemistry on each patient specimen to determine the expression of proteins that frequently undergo alteration in prostate cancer, including PTEN, TMPRSS2-ERG, MYC and Nkx3.1. The results showed that although the clinic-pathological status was similar, there was heterogeneity in the genetic background of patients (Table 1).

**Table 1:** Patient information and genetic background

Patient	PSA at diagnosis (ng/mL)	Gleason score	Pathological Score	BMI	Genetic Background/Status			
					PTEN	MYC	ERG	Nkx3.1
1	3.6	(4 + 3) 7	pT3a	24	Loss	Amplification^1^	Negative	Positive
2	5.8	(3 + 4) 7	pT3a	32	Low expression	Negative	Positive	Positive
3	n/a	(4 + 4) 8	pT3a	29	High expression	Negative	Negative	Positive
4*	6.6	(3 + 4) 7	pT2c	30	Low expression	Negative	Negative	Positive
5*	4.8	(4 + 3) 7	pT3a	26	Low expression	Amplification^1^	Positive	Positive
6*	11.6	(4 + 3) 7	pT3a	26	Low expression	Negative	Negative	Positive

Patients 1–3 were used for study 1 (PDX in HFD and LFD).

Patients 4–6 were used for study 2 (PDX of prostate cancer + PPAT).

* Prostate cancer and PPAT samples were obtained from patients 4–6.

pT2c = Tumour is confined within the prostate but involves both lobes.

pT3a = Tumour has undergone extracapsular extension.

1 Amplification leading to expression detected by immunohistochemistry.

**Figure 2:**
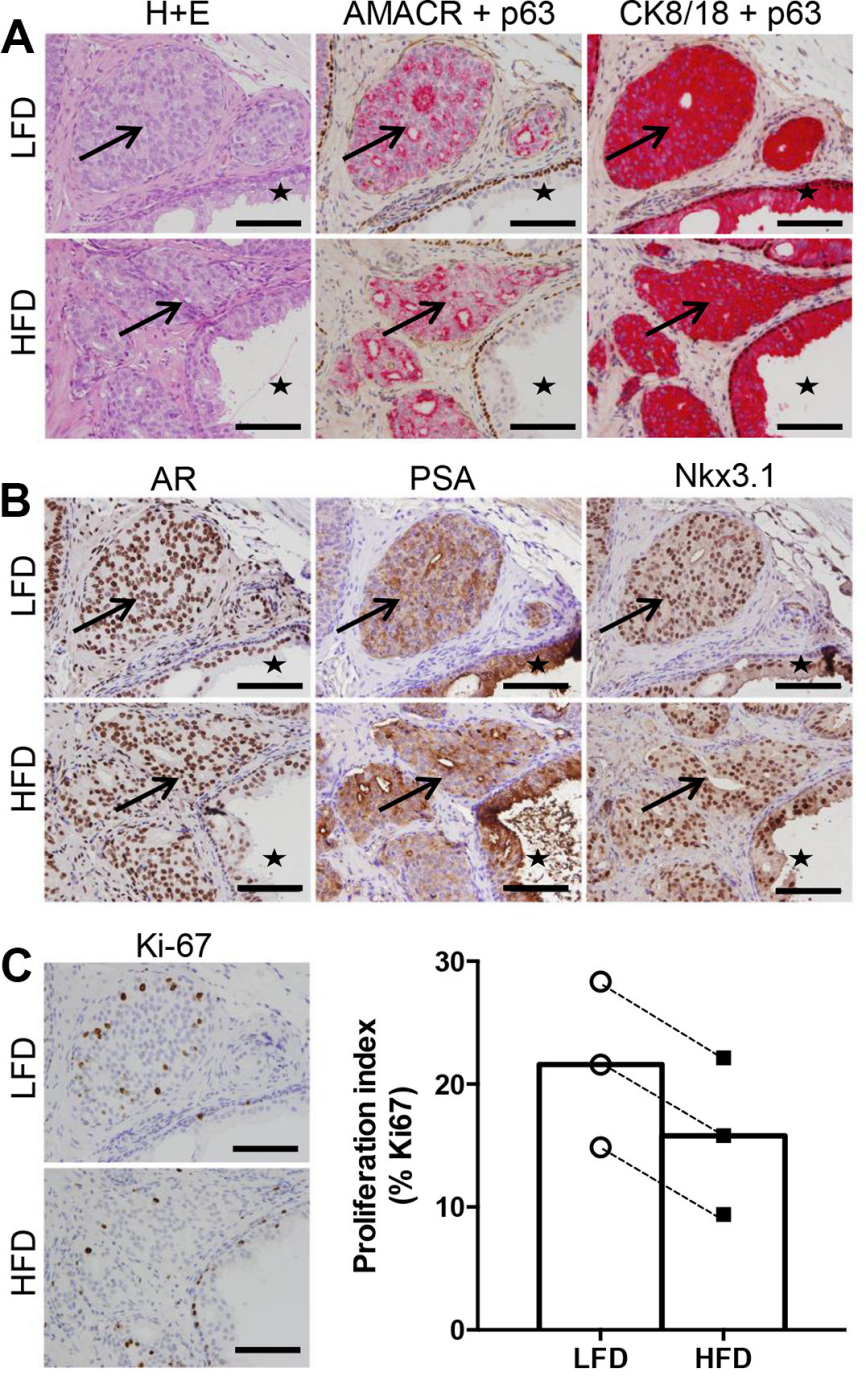
High fat feeding does not promote increased proliferation in patient-derived xenograft tissue (**A**) Haematoxylin and eosin staining, dual immunohistochemistry of AMACR (red) and p63 (brown), cytokeratin 8/18 (red) and p63 (brown). Regions of cancer indicated by arrows, and benign glandular epithelium indicated by star in LFD and HFD xenograft tissue. (**B**) Immunolocalization of AR, PSA and Nkx3.1 in LFD and HFD xenografts. (**C**) Immunolocalization and percentage of Ki67 positive cells in LFD and HFD xenografts (*n* = 6 for LFD and *n* = 6 for HFD). Scale bars 100 μm in all images. Statistical analysis for panel C was by unpaired two-tailed *t*-test. Data are presented as the mean ± SEM.

Prostate cancer specimens were successfully grafted in SCID mice fed both LFD and HFD. Xenografts from all patients contained malignant tumors as shown by H&E pathology, α-methylacyl-CoA racemase (AMACR) expression and loss of p63+ basal cells (Figure 2A). The malignant regions were confirmed to be of human origin based on human-specific cytokeratin 8/18 expression (Figure 2A). Some grafts also contained adjacent benign glands, providing an accurate representation of the heterogeneity of human prostate specimens. Benign regions were excluded from further analysis. In addition, grafts from all patients expressed prostate cancer biomarkers including androgen receptor, PSA, and homeobox protein Nkx3.1 (Figure 2B). Overall, the pathology and biomarker expression of engrafted tissues was similar in the LFD and HFD groups.

The mass and size of grafts harvested from LFD and HFD treatment groups were not different (graft volume: LFD 9.56 ± 1.15 mm^3^ vs HFD 8.24 ± 1.62 mm^3^, *P* = 0.62. Supplementary Figure 1A). The presence of Ki67-positive tumor cells demonstrated malignant foci from all patients were actively proliferating (Figure 2C). Quantitation of Ki67 showed no significant difference between grafts grown in LFD or HFD SCID mice (Figure 2C). Together, these data demonstrate that tumorigenesis of human prostate xenografts was not exacerbated in obese mice.

### Lipidomic analysis reveals no impact of obesity on the tumor lipid mass or composition

**Figure 3:**
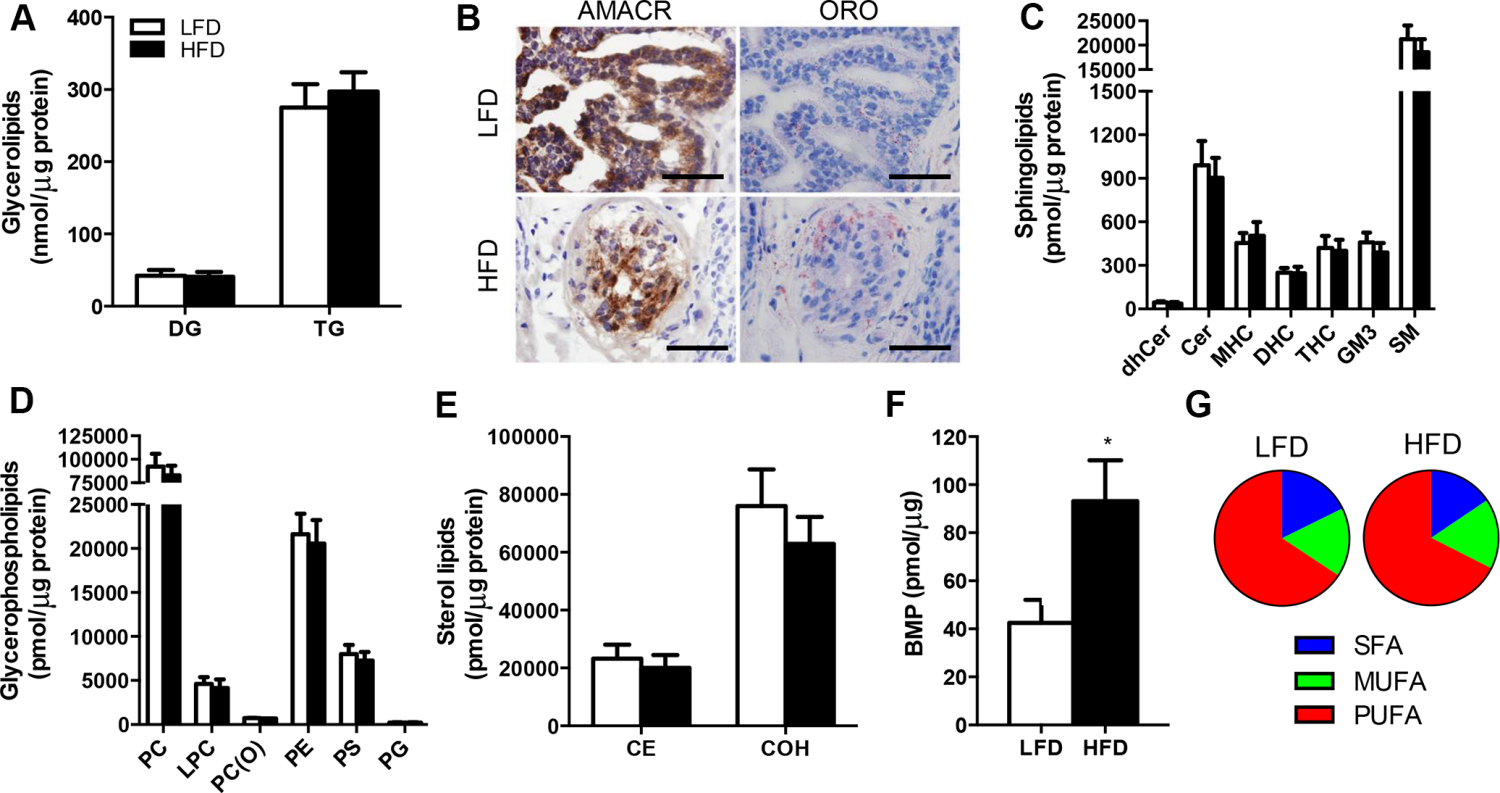
Lipidomic analysis reveals no effect of obesity on lipid deposition in the prostate cancer xenografts in SCID mice (**A**) Glycerolipids in prostate tissue. DG, diglyceride; TG, triglyceride. (**B**) Immunolocalisation of AMACR and Oil Red O staining in malignant foci of LFD and HFD xenografts. Sections were frozen. Scale bars, 50 μm. (**C**) Sphingolipids in prostate tissue. dhCer, dihydroceramide; Cer, ceramide; MHC, monohexosylceramide; DHC, dihexosylceramide; THC, trihexosylceramide; GM3, G_M3_ ganglioside; SM, sphingomyelin. (**D**) Glycerophospholipids in prostate tissue. PC, phosphatidylcholine; LPC, lysophosphatidylcholine; PC(O), alkylphosphatidylcholine; PE, phosphatidylethanolamine; PS, phosphatidylserine; PG, phosphatidylglycerol. (**E**) Sterol lipids in prostate tissue. CE, cholesterol ester; COH, free cholesterol. (**F**) Bis(monoacylglycero)phosphate in prostate tissue. (**G**) Percentage of the prostate lipid pool composed of saturated (SFA), monounsaturated (MUFA) and polyunsaturated (PUFA) fatty acids. For all lipidomic analysis, *n* = 9 for LFD and *n* = 9 for HFD. *P < 0.05 HFD *vs.* LFD. Statistical analysis for individual lipids by unpaired two-tailed *t*-tests. Data are presented as the mean ± SEM.

Alterations in lipid metabolism and remodelling of intracellular lipid pools have been implicated in the pathogenesis of various cancers [22]. Hence, we sought to determine whether obesity induced by high-fat feeding impacts the lipid profile in malignant prostate tissue. Quantitative assessment of the tumor lipidome revealed few significant differences between LFD and HFD mice. There were no differences in glycerolipids including triglycerides and diglycerides (Figure 3A), and these measures were supported by the observation of similar Oil Red O staining of neutral lipids between treatment groups (Figure 3B). The sphingolipid class contains important signalling and structural lipids implicated in cancer pathogenesis, and neither sphingomyelin, hydroxyshpingomyelin, ceramide, monohexosylceramide, dihexosylceramide, trihexosylceramide and dihydroceramide were different in the tumors of LFD and HFD mice (Figure 2C). Glycerophospholipids are critical membrane constituents and their production is required for tumor proliferation and growth [22]. There was no detectable difference in the glycerolphospholipid composition of the tumors between treatment groups (Figure 2D), including the major membrane constituents' phosphatidlycholine, phosphatidylserine and phosphatidylethanolamine (Figure 2D). Similarly, members of the sterol lipids including cholesterol and cholesterol esters were not different between treatment groups (Figure 2E). Of the lipid types assessed, only bis (monoacylglycero) phosphate (BMP) was significantly different between groups, being increased by 120% in tumors from HFD *vs.* LFD mice (Figure 2F). Finally, we examined the proportion of saturated versus unsaturated fatty acids in the tumor lipidome, because increases in dietary saturated fatty acid intake is related to the risk of advanced prostate cancer [23]. The analysis showed that there was no significant differences between saturated (17.7% *vs.* 15.6%), monosaturated (16.5% *vs.* 16.9%) and polyunsaturated lipids (65.7% *vs.* 65.6%) in LFD *vs.* HFD mice (Figure 2G).

### Factors secreted from peri-prostatic adipose tissue promote tumorigenesis in cultured prostate epithelial cells

**Figure 4:**
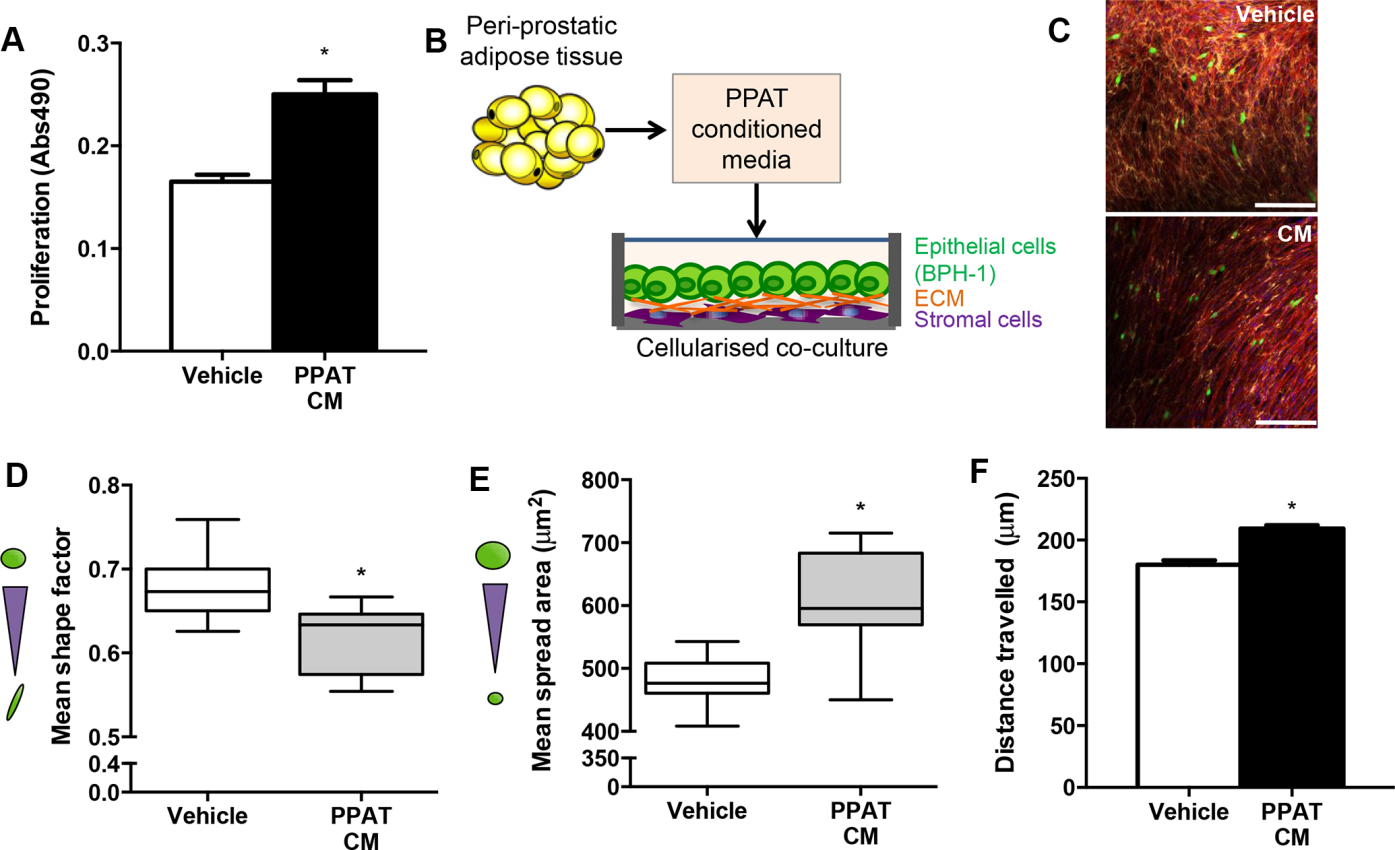
Secreted factors from periprostatic adipose induce a tumorigenic phenotype in a cellularized co-culture model (**A**) Proliferation of PC3 cells grown in vehicle (CHO media alone) compared to vehicle media conditioned with PPAT secreted factors (CM, conditioned media) for 48 hours. Data are presented as the mean ± SEM (*n* = 6 per group from two independent experiments). (**B**) Schematic diagram of experimental design where conditioned media was obtained from human PPAT and applied to a cellularized co-culture model where human primary stromal fibroblasts produce naturally occurring extracellular matrix proteins (ECM) and provide a matrix for BPH-1 cells to be co-cultured for 24 hours. (**C**) Composite image of 3D co-culture components including normal prostatic fibroblasts stained for fibronectin extracellular matrix secretions (yellow), F-actin in fibroblasts (red), BPH-1 cells labelled with cell tracker green and DAPI staining of cell nuclei (blue). Images obtained using immunofluorescent labelling and confocal microscopy. Scale bar = 50 μm. Quantitation of morphological parameters including (**D**) shape factor (measure of elongation; *n* = 7 from independent donors), (**E**) spread area (cell size; *n* = 10 from independent donors) and (**F**) cell migration determined by mean length travelled (*n* = 4 from independent donors, *n* = 184 cells assessed for Vehicle; *n* = 947 cells assessed for PPAT CM). Statistical analysis by unpaired two-tailed *t*-tests. Data are presented as the mean ± SEM.

Given that obesity *per se* did not promote proliferation of human prostate cancer in mice, we reasoned that rather than a systemic mediator, factors secreted from the local PPAT may be important in prostate cancer tumorigenesis. As a proof-of-concept, we assessed whether human PPAT secretes factors that induce a pro-tumorigenic milieu in prostatic epithelial cells. PPAT conditioned medium increased proliferation compared to vehicle in PC3 cells (Figure 4A). We then utilized a bioengineered co-culture model where BPH-1 epithelial cells were grown on a bio-layer of human stromal cells (normal prostatic fibroblasts; Figure 4B showing experimental design and Supplementary Figure 2 showing confocal images). The BPH-1 cells were selected because they are non-tumorigenic but can be permanently transformed by the microenvironment to become tumorigenic [24]. Using the co-culture model, changes in BPH-1 morphology were assessed. PPAT conditioned medium significantly reduced mean shape factor, demonstrating a more elongated phenotype (Figure 4C–4D). Mean spread area, which is a measure of overall cell size, was significantly increased in BPH-1 cells treated with PPAT conditioned media (Figure 4E). Large elongated epithelial cells are consistent with progression to malignancy. Consistent with an invasive phenotype, BPH-1 cells were more motile as indicated by an increase in the mean length that cells travelled (Figure 4F). Together, these data demonstrate that factors secreted from PPAT promote a pro-tumorigenic environment.

### Periprostatic adipose tissue does not influence patient-derived xenograft tumorigenesis in SCID mice

**Figure 5:**
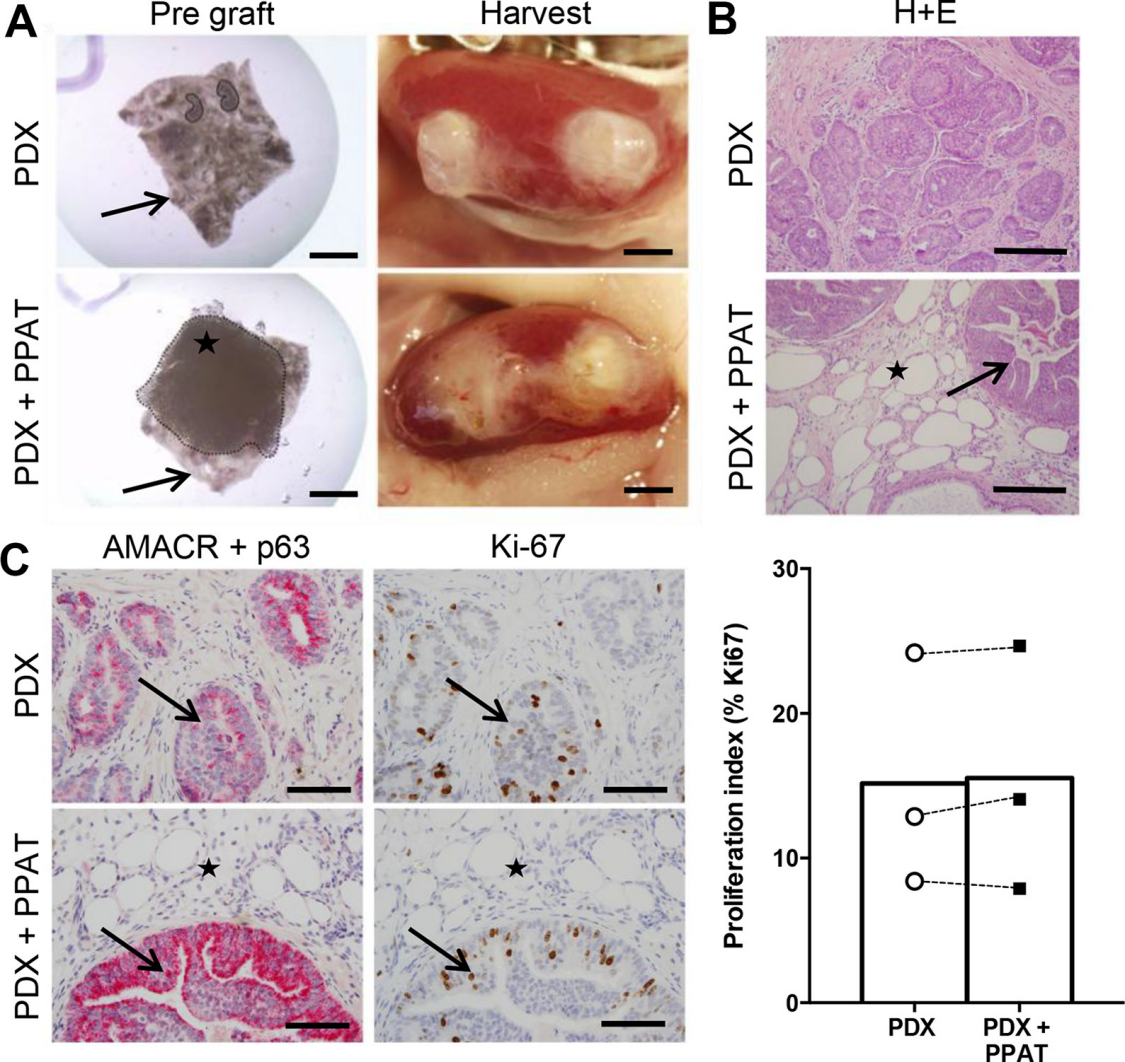
Periprostatic adipose tissue does not promote tumorigenesis in patient-derived xenograft tissues (**A**) Whole mount images of PDX grafts prior to (left panels) and after (right panels) transplantation. PDX grafts contained prostate cancer tissue alone (top panels) or prostate cancer tissue plus human PPAT (bottom panels). Star indicates PPAT spread over the prostate cancer tissue (black arrow). (**B**) Haematoxylin and eosin staining of PDX grafts alone and PDX + PPAT xenografts showing the presence of adipocytes (star) in close proximity to tumor foci (arrow). (**C**) Dual immunohistochemistry of AMACR (red) and p63 (brown) demonstrates malignant foci (arrows) in PDX grafts alone and PDX + PPAT xenografts, with adipocyte infiltration (star) seen in PDX + PPAT tissues. Immunolocalization and quantitation of Ki67 positive cells in PDX grafts alone and PDX + PPAT xenografts. Scale bars, 2 mm (A), 200 μm (B) and 100 μm (C). Statistical analysis for panel C was by unpaired two-tailed *t*-test. Data are presented as the mean ± SEM (*n* = 3).

To investigate the direct effects of human PPAT on prostate cancer proliferation, we co-grafted patient-matched PPAT with prostate cancer tissues (Figure 5A). This approach was used to recapitulate the close interaction between tumor cells and adipose tissue *in vivo* [25]. Visual inspection of harvested grafts confirmed the presence of human PPAT overlaying prostate cancer tissues (Figure 5A) and histological assessment showed the presence of adipocytes adjacent to and within human prostate tissue in co-grafted tissues (Figure 5B). Notably, adipose tissue was not present in prostate cancer only xenografts and there was no evidence of infiltrating adipocytes (Figure 5B). All xenografts contained prostate cancer tissue confirmed by AMACR expression and loss of p63+ basal cells, and there was no obvious difference in pathology between groups (Figure 5C). Consistent with these observations, tumor cell proliferation was not different between groups as demonstrated by quantitation of Ki67 expression (Figure 5C). The graft volume was not different between groups (PDX 8.14 ± 0.86 mm^3^ vs HFD 10.05 ± 0.84 mm^3^, *P* = 0.29. Supplementary Figure 1B).

## DISCUSSION

Large prospective studies report a significant association with obesity for many cancers and obesity is now considered a leading preventable cause of cancer [26, 27]. With respect to prostate cancer, obesity is associated with a small or no increase in prostate cancer incidence but diagnosis of more aggressive, high grade prostate cancer, with poorer prognosis [5]. Despite these associations, there remains a lack of direct evidence linking obesity to accelerated growth of human prostate cancer [25]. Herein, we utilized PDXs of moderate-grade localized human prostate cancer tissues and report two significant findings; that 1) prostate cancer tumorigenicity is not accelerated in the setting of rodent obesity and, 2) PPAT does not exacerbate prostate cancer pathogenesis *in vivo*.

Given the apparent impact of obesity on disease pathogenesis, several studies have attempted to recapitulate the obesity phenotype in rodent models of prostate cancer. These studies utilizing either injectable immortalized human cancer cells [7, 8] or transgenic mice including TRAMP [9, 10, 12], *Pten* [15] and *Hi-Myc* [13] have generally shown that prolonged high-fat feeding accelerates prostate cancer progression as demonstrated by increased tumor volume, proliferation, neoplastic progression, epithelial-mesenchymal transition, metastasis and mortality (see review [25]). While supporting a causative link between obesity and prostate cancer progression, it is notable that these oncogene-driven transgenic mouse models do not faithfully model human prostate cancer development. We sought to overcome these limitations by transplanting localized human prostate cancer [21] into lean and obese mice. We validated the experimental model by demonstrating that SCID mice fed a high-fat diet exhibited the hallmarks of obesity including increased fat mass, fatty liver, fasting hyperglycemia, glucose intolerance, insulin resistance and low-grade inflammation. We combined this obese phenotype with an efficient and reproducible PDX approach that has been optimized to maintain survival and proliferation of human prostate cancer tissues. Notably, we utilized specimens from men with moderate-grade localized prostate cancer rather than high-grade localized or metastatic disease. Using the combination of these validated models and detailed histological analyses, we failed to show that obesity promotes prostate cancer proliferation, at least in these moderate-grade localized human prostate cancer tissues. With respect to study design, the duration of the dietary intervention was comparable to previous studies in transgenic mice (i.e. 10 weeks), and well within the time frame required to modulate proliferation in PDX models. For example, changes in proliferation can be detected within 3 days after castration and 4 weeks after testosterone administration [28, 29], or after 1 week of other androgen-targeted agents, such as Enzalutamide (Taylor, unpublished data).

Obesity is associated with infiltration of immune cells into adipose tissue, which contributes to a state of low-grade inflammation [30], and chronic inflammation is regarded as an enabling characteristic of human cancer [31]. While SCID mice are commonly used for cell and tissue transplantation studies, the absence of mature B and T cells in these mice may pose a potential limitation to the interpretation of our work. However, the pro-inflammatory cytokines implicated in prostate cancer pathogenesis, including IL-6, TNFa and IL-10, were readily detected in serum at concentrations reported in other mouse lines [32], and IL-6 was significantly increased in obese compared with lean mice. This is consistent with the small, but significant changes that occur for most circulating pro-inflammatory cytokines when transitioning from the lean to obese state (i.e. < 5 pg/mL). In prostate cancer, the levels of serum IL-6 range from 1.5–2.2 pg/mL in localized and locally invaded tumors, which is well below the serum levels reported (~100 pg/mL) when metastatic disease ensues [33, 34]. Moreover, IL-6 is produced by prostatic stromal cells, including endothelial cells and macrophages, and appears to act in a paracrine manner in prostate tissue [35]. Thus, the modest obesity-induced pro-inflammatory milieu, involving small increases in systemic IL-6, are unlikely to impact the local IL-6 concentrations and accelerate inflammation-driven cancer progression. In addition, other pro-inflammatory cytokines/chemokines including TNFa, MCP-1, IL-8, or IL-1ß are not increased in prostate cancer patients. On balance, these observations suggest that obesity-associated inflammation is not a primary driver of prostate cancer progression.

It has been increasingly recognized that alterations in lipid metabolism are a hallmark of cancer [22, 31] and some evidence indicates that prostate cancer is associated with an increased reliance of fatty acid as a metabolic fuel and as a precursor for phospholipid production to generate cell membranes [36, 37]. Accordingly, we aimed to define the lipid profile of human prostate cancer tissues and hypothesized that obesity would alter the lipidome, potentially identifying a signature that might be prognostic for aggressive disease. Unbiased mass spectrometry analysis revealed no significant differences in the quantity of lipids (with the exception of Bis (Monoacylglycero)Phosphate), the type of lipids species detected or the degree of saturation within the prostatic lipid pool in prostate cancer PDX grafts from lean and obese mice. These results were surprising because the uptake and storage of fatty acids in almost all tissues is considered a pathogenic feature of obesity [38]. While lipid kinetics were not assessed in our studies, these results indicate that fatty acid uptake and storage are unaffected, and/or oxidative disposal of fatty acid is increased in the prostate PDX grafts in obesity. Further studies are needed to clarify whether changes in lipid metabolism are related to prostate cancer progression.

The only lipid that was increased in PDX grafts from HFD compared to LFD mice was BMP, an anionic phospholipid found in the lysosomal membrane. BMP contains a large quantity of hydrolases, known as cathepsins, which have been implicated in human cancer metastasis to breast and lung cancer [39, 40]. Whether alterations in BMP are important in prostate cancer is unknown, but given the similarity in the proliferation index in PDX grafts from lean and obese mice, it is unlikely that small changes in BMP are sufficient to drive prostate cancer progression.

Aside from obesity-driven systemic effects, it is also possible that secreted factors from the local PPAT influence the prostatic milieu to direct prostate cancer growth, invasion and possibly distant metastases. This view is based on the clinical observation that PPAT quantity is increased in obesity and is a risk factor for both the diagnosis of prostate cancer and predicting high-grade disease [41, 42]. In addition, *in vitro* studies have reported that factors secreted from PPAT increase proliferation and motility in PC3 prostate cancer cells, and that the PPAT-secreted factors from obese patients are more pro-tumorigenic than those from lean patients [18, 43]. We confirmed and extended on the key results of these *in vitro* studies by demonstrating pro-tumorigenic effects of PPAT-secreted factors in a cellularized co-culture model that incorporates human primary stromal-epithelial interactions, which more closely mimics the tumor microenvironment [24]. Ongoing studies are delineating the bioactive factors secreted from PPAT that may influence prostate cancer progression.

In order to understand the relevance of PPAT in promoting prostate cancer pathogenesis *in vivo*, we co-grafted human PPAT with prostate cancer tissues to mimic the prostate/PPAT anatomical relationship. Histological analysis showed successful harvest of both tissue types, evidence of adipocyte infiltration into the prostatic tissue and direct contact between cancer cells and adipocytes, mimicking an aggressive stage of disease involving local invasion [25]. Despite the knowledge that the PPAT secreted milieu is pro-tumorigenic in culture, we saw no evidence of increased proliferation in the prostate cancer grafts grown in association with PPAT compared to prostate cancer tissues grafted alone. Taken together, our data indicate that PPAT secreted factors can promote prostate cancer tumorigenesis *in vitro*, however, the secretion rates *in vivo* and the potential for dilution of PPAT-secreted factors through the systemic circulation question the absolute effect *in vivo*.

In conclusion, it is now 75 years since the initial observation that high-fat feeding induces obesity and promotes cutaneous tumor formation in mice [44]. While the epidemiological evidence indicates that obesity is associated with worse outcomes for prostate cancer patients, our data question a causative role for obesity in driving prostate cancer proliferation in moderate-grade localized disease, which is a common form of prostate cancer. However, we cannot discount that obesity may exacerbate tumorigenesis in high-grade prostate cancers, and this will be the subject of future investigations.

## MATERIALS AND METHODS

### Human ethics approval and collection of human tissues

Ethics approval was obtained from Cabrini Institute (07-07-04-14), Epworth Hospital (618-13) and Monash University Human Research Ethics Committees (RMO 2006/6108 – 2004000145). Patients provided informed written consent to medical treatment reports and the collection of fresh prostate tumor tissue. Treatment notes were accessed through physicians, hospitals and diagnostic laboratories. Fresh primary prostate cancer tissues were obtained from six patients at the time of radical prostatectomy. All patients had moderate-grade prostate cancer based on Gleason grade, prostate-specific antigen (PSA) and pathological stage (Table 1). The body mass index of patients averaged 28 ± 1 kg/m^2^, placing them in the overweight range. Pathologists dissected a tumor region for this study, which was subsequently transported to the laboratory in RPM1 1640 supplemented with 10% fetal calf serum (FCS), 1% penicillin-streptomycin, 0.5 μg/ml amphotericin B antimycin and 100 μg/ml gentamicin. For studies utilizing peri-prostatic adipose tissue (PPAT), patient matched adipose tissue samples were collected at the time of radical prostatectomy (patients 4–6; Table 1). PPAT was transported to the laboratory in RPMI media (Gibco, CA, USA) supplemented with 5 mM glucose (Sigma-Aldrich, MI, USA), 500 nM adenosine (Sigma-Aldrich, MI, USA) and 10 mg/ml of fatty acid free bovine serum albumin (Bovogen Biologicals, Australia).

### Animal ethics and experimental design

All procedures were approved by The Monash Animal Research Platform (MARP) Animal Ethics Committee (MARP/2012/158) and were performed in accordance with Australian National Health and Medical Research Council Guidelines on Ethics in Animal Experimentation. Severe combined immunodeficiency (SCID) mice were obtained from the Animal Resources Centre (Perth, Australia) and were housed three mice per cage. Mice were kept on a 12-hour light, dark cycle with *ad libitum* access to food and water. Mice were either fed a standard low-fat diet (LFD, *n* = 12) (6% energy from fat, 20% protein, 74% carbohydrate; Barastoc, Ridley Corporation, Melbourne, Victoria, Australia) or a high-fat diet (HFD, *n* = 12) (43% energy from fat, 21% protein, 36% carbohydrate; SF04-001, Speciality Feeds) starting at five weeks of age. There were subtle differences in the macronutrient content of the diet, most notably the protein source, which was derived from casein in the HFD and from several sources in the LFD. The vitamin and mineral contents of the diets were similar (Supplementary Table 1). Mice were maintained on these diets for six weeks prior to PDX surgery. Mice aged 11 weeks underwent PDX surgery and were maintained on their respective diets for a further 10 weeks. In experiments designed to assess the effects of PPAT on human prostate cancer proliferation, all mice were fed a standard LFD. Body mass was monitored bi-weekly, glucose tolerance was assessed two weeks prior to PDX surgery and 10 weeks following surgery, and body composition was assessed two days prior to surgery (Figure 1A).

### Patient-derived xenografts

Grafts were implanted under the kidney capsule of SCID mice as described previously [21]. Briefly, prostate specimens were sliced into 300 μm sections using the Krumdieck tissue slicer (Model number MD6000, Alabama Research and Development, AL, USA), with approximately every 5th section being fixed in formalin for pre-grafting histology. The remaining tissue slices were stored in RPMI/10% FCS/10 nM testosterone at 4°C until PDX preparation. Systematic precision-cutting of tissue slices allowed for pair-matched analysis between pre-grafted tissues, and PDX groups, thereby accounting for differences in tumor composition between individual pieces. Prostate tissue slices were combined with ~250,000 mouse seminal vesicle mesenchyme cells to aid the survival and growth of prostate PDXs *in vivo*. The seminal vesicle mesenchyme was isolated from day 0 BALB/c male mouse pups (Monash Animal Services). Xenografts were transplanted under the kidney capsule of host SCID mice for 10 weeks. Each host mouse was implanted with two grafts per kidney. At the time of graft surgery, host mice were implanted with a 5 mm testosterone implant to supplement the host testosterone levels. For analysis of the effects of a LFD or HFD on prostate cancer proliferation, three individual prostate donor tissues were utilized, and grafts were evenly divided between three LFD and three HFD SCID mice per patient.

For PPAT studies, PDX tissues were grafted as previously described with minor modifications [21]. Specifically, half of the PDX tissues were prepared without PPAT while the other half of the PDX tissues were combined with pieces of patient matched PPAT tissue (~ 4 mm × 4 mm × 1 mm pieces) prior to renal grafting. Adipose tissue was placed directly over the prostate tissue within the collagen gel as shown in Figure 5A, and the collagen gel containing the tissues was grafted under the kidney capsule. The PDX tissues without PPAT were grafted under the capsule of the contralateral kidney. For analysis of PPAT effects on prostate cancer proliferation, a further three patient tissues were utilized, with PDX tissue alone or PDX + PPAT grafts produced for each patient.

### Glucose tolerance test

Mice were fasted for four hours (0700–1100 h) then injected with D-glucose (2 g/kg body mass) in the intraperitoneal cavity. Blood glucose was measured using a glucometer (Accu-chek, Roche, Mannheim, Germany) before and at 15 min intervals for 120 min after glucose administration.

### Assessment of body composition

Body composition was analyzed using Dual Energy X-ray absorptiometry (DEXA; Lunar Pixi, PIXImus, WI, USA) as described previously [45].

### Tissue collection

Ten weeks after PDX surgery, mice were fasted for four hours (0700–1100 h) then euthanized via cervical dislocation. Blood was collected by cardiac puncture, placed in an ethylenediaminetetraacetic acid collection tube and left of ice for 5 mins. Plasma was separated by centrifugation at 8000 × *g* for 10 mins and stored at −80°C until analysis. The livers of mice were dissected and rapidly frozen in liquid N_2_ for later analysis. Prostate cancer xenografts were removed from the kidney grafting site, with three grafts per mouse rapidly frozen in liquid N_2_ for later lipid analysis. The remaining graft per mouse was fixed in 10% neutral buffered formalin (Sigma-Aldrich, MA, USA) for 24 h before being processed and embedded in paraffin (*n* = 3 per patient/per diet group) for histological analysis.

### Histological analysis

PDX tissues were sectioned (5 μM) and every 20th tissue section was used for analysis. Immunohistochemistry analysis was performed using the Leica BOND-MAX automated system (Leica Microsystems, Victoria, Australia) or manually using the Dako EnVision™ detection system (Dako, California, USA). Primary and secondary antibody details and staining conditions are outlined in Supplementary Table 2. Ki67 immunohistochemical staining was performed on three representative sections from each fixed xenograft. Slides were scanned using the Aperio ScanScope AT Turbo (Leica Microsystems GmbH, Germany) and positive cells were determined using Aperio ImageScope analysis software (version 12.2).

### Plasma hormones and metabolites

Plasma insulin was determined using an Ultra-Sensitive Mouse Insulin ELISA Kit (Crystalchem, IL, USA) according to manufacturer's instructions. Plasma free fatty acid concentration was determined using an enzymatic colorimetric assay (NEFA C Kit; Wako Chemicals, Richmond, VA, USA). Plasma triglyceride levels were determined using a colorimetric assay (Triglycerides GPOPAP; Roche Diagnostics, IN, USA).

### Liver triglyceride content

Lipids were extracted from ~20 mg of liver in chloroform: methanol: PBS + 0.2% SDS (1:2:0.8) using a handheld homogenizer (Pro Scientific, Oxford, CT, USA). After brief centrifugation (1,000 × *g* for 10 min), the organic phase containing the lipids was transferred to a fresh tube and dried under N_2_ at 40^°^C. The dried lipid was reconstituted in ethanol and the triglyceride levels were determined using the triglyceride GPOPAP assay (Roche Diagnostics, IN, USA).

### Lipid extraction and MS analysis

Lipid extraction and MS analysis was performed as described previously [46]. In brief, prostate cancer xenografts were harvested and stored at −80°C prior to lipid extractions. The whole prostate cancer xenograft was homogenized in ice-cold 1 X PBS and lipids were extracted using chloroform: methanol (2:1). Samples were dried and reconstituted in water saturated butanol and methanol followed by the addition of internal standards in chloroform:methanol (1:1, 15 μl). Lipid analysis was performed on the samples by liquid chromatography electrospray ionization-mass spectrometry/mass spectrometry using an Agilent 1200 liquid chromatography system (Agilent, CA, USA) and Applied Biosystems API 4000 Q/TRAP mass spectrometer with a turbo-ionspray source (Applied Biosystems, CA, USA) and Analyst 1.5 and Multiquant data systems (Sciex, MA, USA). Quantitative analysis of individual lipid species was performed using Multiquant v1.2 and the cellular content of each lipid class was calculated by summing each lipid species within that class.

### PPAT conditioned media preparation

For conditioned media collection, approximately 1–3 g of fresh PPAT tissue was diced into ~25 mg pieces and incubated in EX-CELL™ 325 PF CHO Serum-Free Medium, Protein Free (Sigma-Aldrich, MI, USA) supplemented with 5 mM glucose (Sigma-Aldrich, MI, USA), protease inhibitor cocktail (Roche, Bavaria, Germany) and 500 nM adenosine (Sigma-Aldrich, MI, USA) for 5 hours at 37°C in 5% CO_2_. Prior to use for cell culture, conditioned media was passed through a 0.22 μm filter and supplemented with 5% heat inactivated fetal calf serum (Gibco, CA, USA), antibiotics (10,000 U/mL penicillin and 10,000 μg/mL streptomycin; Gibco, CA, USA) and 1 nM testosterone (Sigma-Aldrich, MI, USA).

### *In vitro* co-culture

Primary normal prostate fibroblasts were isolated from prostate specimens [21, 47] and co-culture experiments were performed as previously described. Briefly, fibroblasts were seeded at 1.5 × 10^4 i^n 24 well plates and were grown in RPMI media (School of Biomedical Sciences, Media Prep Services, Monash University, Victoria, Australia) supplemented with 5% FCS (Gibco, CA, USA), antibiotics (10,000 U/ml penicillin and 10,000 μg/mL streptomycin, Gibco, CA, USA), 1 nM testosterone (Sigma-Aldrich, MI, USA) and 10 ng/mL FGF (Millipore, MA, USA) at 37°C, 5% CO_2 f_or approximately 7 days to allow confluency and secretion of extracellular matrix. Then 1.5 × 10^4^ BPH-1 epithelial cells (obtained from S. Hayward, Vanderbilt), pre-stained with Cell Tracker green CMFDA (5-chloromethylfluorescein diacetate; ThermoFisher Molecular Probes, MA, USA) were seeded on top of the fibroblasts. At this time, co- culture media was changed to PPAT conditioned media and cells were cultured for a further 24 hours at 37°C, 5% CO_2._

### Immunofluorescence staining

Matrix proteins were visualized using immunofluorescence for both fibronectin (ECM) and phalloidin (F-actin). To permeabilize cell membranes, cells were washed with 0.1% Triton X-100 (BDH Prolabo, Queensland). To reduce non-specific binding, cells were incubated with 1% bovine serum albumin (BSA; Sigma-Aldrich, MO, USA) for 10 min at room temperature before incubation in anti-fibronectin mouse monoclonal antibody (HFN7.1; 1:200 dilution) (Abcam, UK) at room temperature for 40 min. Cells were incubated with secondary antibodies for 30 min at room temperature. These included goat anti-mouse isotype specific IgG1 antibody tagged with alexa fluro 647 (Invitrogen Molecular Probes, California, USA; 1:400 dilution) for fibronectin, rhodamine phalloidin (Invitrogen Molecular Probes, California, USA; 1:300 dilution) to detect actin filaments, and DAPI (Invitrogen Molecular Probes, California, USA) to visualize cell nuclei. Cells were then washed with PBS and left at 4^°^C in PBS until imaging.

### Morphological analysis

Co-culture plates were imaged using a Leica DM IL LED inverted fluorescent microscope (Leica Microsystems, Victoria, Australia). Assays were performed in duplicate with three technical replicates per well and analyzed as the combined mean of six images per fibroblast line. Quantitative analysis of BPH-1 cell morphology was performed using ImageJ software [48]. Parameters analyzed included shape factor, spread area and mean length travelled, which provides a measure of motility.

### Proliferation assay

For proliferation assays, 3 × 10^3^ PC3 cells (ATCC, VA, USA) were seeded into 96 well plates and incubated in Vehicle or PPAT conditioned media for 48 hours. The “Vehicle” medium contained EX-CELL™ 325 PF CHO Serum-Free Medium, Protein Free medium supplemented with 5% heat inactivated fetal calf serum and antibiotics. The “Conditioned Medium” consisted of the same base ingredients plus PPAT secreted factors (described above). The Cell Titre 96 Aqueous One assay (Promega, WI, USA) was used to assess cell proliferation and absorbance was measured at 490 nm using a FLUOstar Optima plate reader (BMG LABTECH, Baden-Wüttemberg, Germany).

### Statistical analysis

All data presented as means ± SEM. Data were analyzed by two-tailed unpaired student's *t*-tests or two-way repeated measures analysis of variance, with a Bonferroni multiple comparisons post-hoc test where appropriate. Statistical significance was determined *a priori* at *P* < 0.05.

## SUPPLEMENTARY MATERIALS


