# Supplementary material for: Obesity does not promote tumorigenesis of localized patient-derived prostate cancer xenografts
Source: Oncotarget. 2016 Jun 23;7(30):47650–62. doi: 10.18632/oncotarget.10258 (PMC5216968; doi:10.18632/oncotarget.10258)
Supplement: Supplementary file 1 [file oncotarget-07-47650-s001.pdf]

Obesity does not promote tumorigenesis of localized patient-derived prostate cancer xenografts

Supplementary Materials

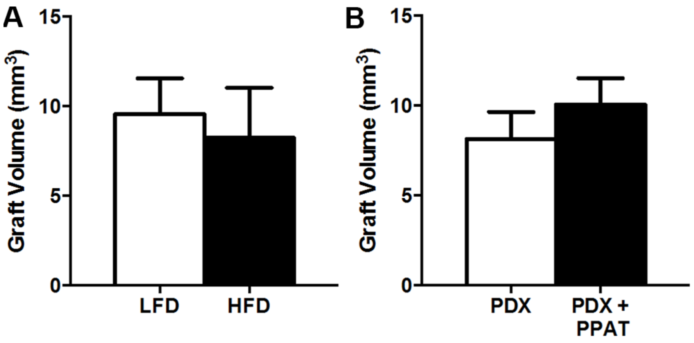

Supplementary Figure S1: Graft volume of PDXs. Graft volumes of harvested PDXs obtained from mice. (**A**) LFD vs. HFD (n = 3 patients per group) and (**B**) PDX alone vs. PDX + PPAT (n = 3 patients per group). Data are shown as mean ± SEM.

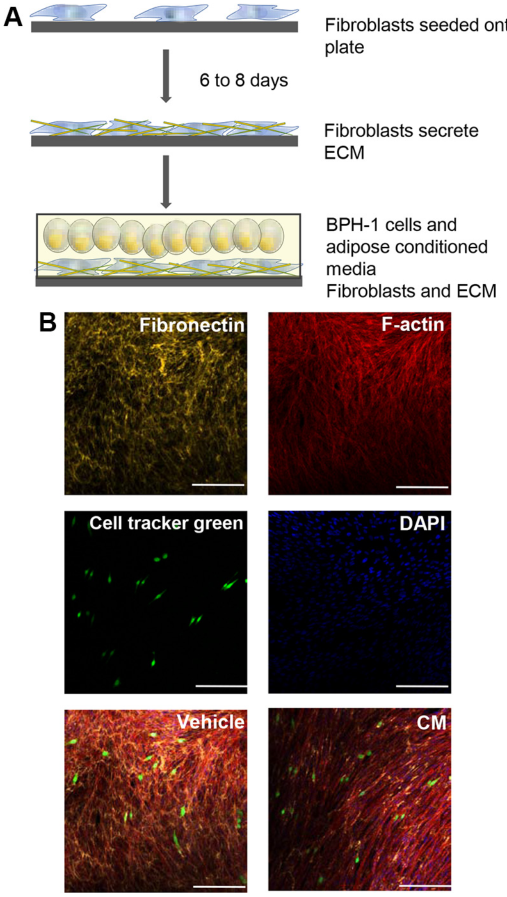

Supplementary Figure S2: Establishment of the cellularized matrix in 3D co-culture model. (**A**) Schematic representation of the co-culture model. Human primary stromal fibroblasts (normal prostatic fibroblasts) are plated and allowed to produce naturally occurring extracellular matrix proteins over 6–8 days. BPH-1 cells stained with cell tracker green dye are then culture on the stromal – matrix layer for 24 hours. PPAT condition media is added to the culture wells. (**B**) Immunofluorescent labelling of 3D co-culture components. Fibronectin staining of extracellular matrix secretions, immunofluorescent labelling of F-actin in fibroblasts, BPH-1 cells labelled with cell tracker green and DAPI staining of cell nuclei. Composite image of BPH-1 cells co cultured on cellularised matrix in vehicle media alone and with adipose conditioned media. Images are representative of all co-cultured. Scale bar = 50 μm (B).

Supplementary Table S1: Dietary information. See Supplementary_Table_S1

Supplementary Table S2: Details of primary and secondary antibody used for immunohistochemistry staining

Primary Antibody	Clone	Supplier	Species and isotype	Concentration used
**AMACR**	13H4	DAKO	Rabbit IgG	0.55 μg/ml
**AR**	A9853	Sigma Aldrich	Rabbit IgG	2.0 μg/ml
**CK8/18**	NCL-L-5D3	Novocastra	Mouse IgG_1	0.26 μg/ml
**ERG**	EPR3864	AbCAM	Rabbit IgG	3.26 μg/ml
**Ki-67**	MM1	Novocastra	Mouse IgG_1	0.2 μg/ml
**MYC**	Y69	AbCAM	Rabbit IgG	0.19 μg/ml
**Nkx3.1**	-	Athena Enzyme Systems	Rabbit IgG	0.67 μg/ml
**p63**	-	AbCAM	Rabbit IgG	0.25 μg/ml
**PSA**	A0562	DAKO	Rabbit IgG	1 μg/ml
**PTEN**	-	Cell Signalling	Rabbit IgG	0.06 μg/ml
